# Hyperbilirubinemia After Redo Valve Surgery: Incidence, Perioperative Risk Factors, and Association with Early Clinical Outcomes

**DOI:** 10.3390/jcdd13060268

**Published:** 2026-06-15

**Authors:** Can Zhao, Wei Yao, Jianping Xu, Guangyu Pan, Shen Liu

**Affiliations:** 1 Department of Cardiac Surgery, Peking University International Hospital, Life Park Road No.1 Life Science Park of Zhong Guancun, Changping District, Beijing 102206, China; 2 Department of Cardiac Surgery, Peking University People’s Hospital, Beijing 100044, China

**Keywords:** cardiopulmonary bypass, redo valve surgery, hyperbilirubinemia, risk factors

## Abstract

Background: Postoperative hyperbilirubinemia is a serious complication after cardiac surgery and has been associated with increased perioperative morbidity and mortality. However, data specifically addressing patients undergoing redo valve surgery remain limited. This study aimed to determine the incidence and risk factors of postoperative hyperbilirubinemia after redo valve surgery, and evaluate its association with early postoperative outcomes. Methods: We retrospectively reviewed 259 adult patients who underwent elective redo valve surgery under cardiopulmonary bypass (CPB) between March 2018 and July 2024. Postoperative hyperbilirubinemia was defined as a serum total bilirubin level > 3 mg/dL at any time after surgery. Patients were divided into a hyperbilirubinemia group and a non-hyperbilirubinemia group. Perioperative variables were compared between groups. Univariable and multivariable logistic regression analyses were performed to identify risk factors for postoperative hyperbilirubinemia. Postoperative complications and in-hospital mortality were also compared. Results: Postoperative hyperbilirubinemia occurred in 101 of 259 patients (39.0%). Compared with patients without hyperbilirubinemia, those with hyperbilirubinemia had longer mechanical ventilation and intensive care unit stay, and higher rates of pneumonia, reintubation, tracheostomy, continuous renal replacement therapy, and in-hospital mortality. Univariable logistic regression showed that higher EuroSCORE II, higher preoperative total bilirubin and direct bilirubin levels, lower hemoglobin and platelet count, pulmonary hypertension, anemia, longer operative time, CPB duration, and aortic cross-clamp time, lower nasopharyngeal temperature, greater intraoperative blood loss, larger red blood cell and plasma transfusion volumes, and concomitant surgery on all three valves were associated with postoperative hyperbilirubinemia. Multivariable analysis identified elevated preoperative direct bilirubin, prolonged CPB duration, and more plasma transfusion as independent risk factors. Receiver operating characteristic analysis showed that peak postoperative total bilirubin had moderate prognostic discrimination for in-hospital mortality, with an optimal cut-off value of 3.95 mg/dL (AUC 0.756, sensitivity 66.7%, specificity 80.2%, *p* = 0.003). Conclusions: Postoperative hyperbilirubinemia is common after redo valve surgery and is associated with worse early postoperative outcomes and higher in-hospital mortality. In this setting, postoperative bilirubin elevation should be interpreted primarily as a prognostic marker of perioperative stress and hepatic vulnerability rather than a direct causal driver of adverse outcomes. Elevated preoperative direct bilirubin, prolonged CPB duration, and greater plasma transfusion were independently associated with the development of postoperative hyperbilirubinemia in this high-risk population.

## 1. Introduction

With the aging population, increasing life expectancy, advances in surgical techniques, and broader use of bioprosthetic valves, the number of patients requiring redo valve surgery has steadily increased [1,2,3,4,5]. Despite continuous improvements in perioperative management, including cardiopulmonary bypass technology, myocardial protection, cardiac anesthesia, surgical techniques, and postoperative intensive care, redo valve surgery remains associated with substantial perioperative risk and mortality [6]. Postoperative hyperbilirubinemia is one of the major complications after cardiac surgery and has been linked to increased mortality [7,8]. Because of differences in surgical procedures and definitions of hyperbilirubinemia, the reported incidence varies widely, ranging from 3% to 57% [9,10,11]. Previous studies suggest that the incidence of hyperbilirubinemia is higher after valve surgery than after coronary artery bypass grafting or congenital heart surgery [8,12,13]. However, studies focusing specifically on redo valve surgery are scarce. Therefore, the present study aimed to clarify the incidence of postoperative hyperbilirubinemia after redo valve surgery, identify its perioperative risk factors, and evaluate its clinical significance with respect to early postoperative morbidity and mortality.

## 2. Materials and Methods

### 2.1. Study Design and Population

This retrospective observational study was approved by the Ethical Review Committee of Peking University International Hospital (Beijing, China). Written informed consent was obtained from all patients according to institutional requirements.

We included consecutive adult patients who underwent elective redo valve surgery under cardiopulmonary bypass in the Department of Cardiac Surgery, Peking University International Hospital, between March 2018 and July 2024. The inclusion criteria were: (1) elective surgery; (2) history of previous valve surgery; and (3) redo valve surgery performed under CPB. The exclusion criteria were: (1) age < 18 years; (2) emergency surgery; and (3) active hepatitis. A total of 259 patients were included and divided into two groups according to the serum total bilirubin level.

### 2.2. Data Collection and Definitions

Clinical data were extracted from the electronic medical record system. Baseline variables included age, gender, body mass index (BMI), interval between the two operations, diabetes mellitus, hypertension, smoking history, pulmonary hypertension, atrial fibrillation, anemia, and infective endocarditis. Cardiac functional parameters, including left ventricular ejection fraction (LVEF) and New York Heart Association (NYHA) status, were recorded. EuroSCORE II was calculated for all patients. Preoperative laboratory data included total bilirubin, direct bilirubin, serum creatinine, estimated glomerular filtration rate (eGFR), albumin, alanine aminotransferase, aspartate aminotransferase, hemoglobin, white blood cell count, and platelet count. Postoperative total bilirubin and direct bilirubin levels, as well as the time to peak postoperative total bilirubin, were also recorded. Operative variables included total operative time, CPB duration, aortic cross-clamp time, lowest intraoperative nasopharyngeal temperature, mean blood pressure during CPB, type of surgical procedure, intraoperative blood loss, and volumes of red blood cell, plasma, and platelet transfusion during surgery and within the first 48 postoperative hours. Postoperative outcomes included duration of mechanical ventilation, intensive care unit (ICU) length of stay, and postoperative hospital length of stay. Postoperative complications included pneumonia, reintubation, tracheostomy, renal replacement therapy, extracorporeal membrane oxygenation (ECMO) use, and in-hospital mortality.

According to commonly used criteria in the literature, postoperative hyperbilirubinemia was defined as a serum total bilirubin level > 3 mg/dL at any time after surgery. Preoperative hyperbilirubinemia was defined as a preoperative total bilirubin level > 2 mg/dL.

### 2.3. Statistical Analysis

Statistical analysis was performed using SPSS version 26.0 (IBM Corp., Armonk, NY, USA). Continuous variables are presented as median (interquartile range) or mean ± standard deviation, as appropriate, and categorical variables as number (percentage). Continuous variables were compared using Student’s *t*-test or the Mann–Whitney U test, depending on distribution. Categorical variables were compared using the chi-square test or Fisher’s exact test. Potential risk factors for postoperative hyperbilirubinemia were first assessed using univariable logistic regression. Candidate variables for the multivariable logistic regression model were selected based on both clinical relevance and univariable associations. To reduce overfitting and collinearity, highly correlated operative variables were not entered simultaneously. In particular, because CPB duration showed substantial collinearity with total operative time and aortic cross-clamp time, only CPB duration was retained in the final multivariable model as the operative variable most representative of procedural complexity and exposure to extracorporeal circulation. A forward stepwise method based on maximum likelihood estimation was used. A two-sided *p* < 0.05 was considered statistically significant. Receiver operating characteristic (ROC) analysis was performed to evaluate the predictive value of peak postoperative total bilirubin for in-hospital mortality. The optimal cut-off value was determined using the Youden index.

## 3. Results

### 3.1. Patient Characteristics and Incidence of Postoperative Hyperbilirubinemia

A total of 259 patients were included in the study. The mean age was 55.9 ± 13.5 years (range, 18–79 years), and 160 patients (61.78%) were female. The surgical procedures included redo aortic valve surgery in 10 patients, redo mitral valve surgery in 33, redo tricuspid valve surgery in 36, redo aortic plus mitral valve surgery in 16, redo aortic plus tricuspid valve surgery in 24, redo mitral plus tricuspid valve surgery in 71, and redo surgery involving the aortic, mitral, and tricuspid valves in 69. In addition, 7 patients underwent concomitant coronary artery bypass grafting. The mean interval between the two operations was 16.1 ± 9.2 years. During hospitalization, 12 patients died, 11 developed pneumonia, 14 required reintubation, 8 underwent tracheostomy, 21 required in-hospital dialysis, and 1 patient received ECMO support.

**Figure 1:**
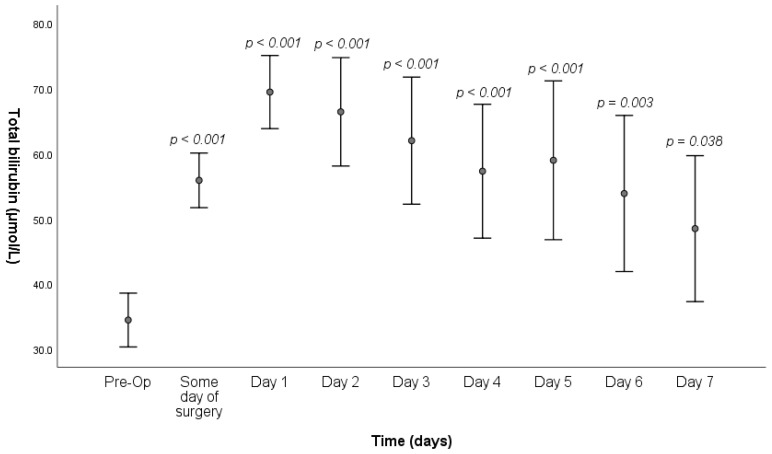
Serial serum total bilirubin concentrations during the first 7 postoperative days in patients with postoperative hyperbilirubinemia. Compared with preoperative baseline level, serum bilirubin levels at all postoperative time points within 7 days were significantly elevated (all *p* < 0.05). Most patients reached peak bilirubin levels within the early postoperative period, supporting close bilirubin monitoring during the first 72 h after surgery. The observed trend is more likely to reflect perioperative stress and hepatobiliary dysfunction than a single causal mechanism.

Postoperative hyperbilirubinemia occurred in 101 patients, corresponding to an incidence of 39.00% (101/259). The predominant pattern was direct hyperbilirubinemia. Among patients with postoperative hyperbilirubinemia, 60 (59.41%) had a direct bilirubin/total bilirubin ratio >50%, with 7 deaths; 39 patients (38.61%) had a ratio between 20% and 50%, with 2 deaths; and 2 patients (1.98%) had a ratio <20%, with no deaths. Among the 101 patients with postoperative hyperbilirubinemia, peak total bilirubin occurred on the day of surgery in 18 patients (17.82%, 1 death), on postoperative day 1 in 35 patients (34.65%, 2 deaths), day 2 in 22 patients (21.78%, 4 deaths), and day 7 in 3 patients (2.97%, 1 death). Changes in total bilirubin during the first 7 postoperative days are shown in Figure 1. Patients were divided into the hyperbilirubinemia group and the non-hyperbilirubinemia group according to postoperative total bilirubin levels. Baseline and perioperative characteristics are summarized in Table 1.

### 3.2. Comparison of Preoperative and Intraoperative Characteristics

Patients in the hyperbilirubinemia group had significantly higher rates of preoperative pulmonary hypertension and anemia than those in the non-hyperbilirubinemia group (*p* = 0.003 and *p* = 0.006, respectively). They also had significantly higher preoperative EuroSCORE II, total bilirubin, direct bilirubin, and aspartate aminotransferase levels (all *p* < 0.05), whereas hemoglobin and platelet count were significantly lower (*p* = 0.014 and *p* = 0.015, respectively). With respect to intraoperative variables, the hyperbilirubinemia group had significantly longer operative time, CPB duration, and aortic cross-clamp time, greater intraoperative blood loss, and more red blood cell and plasma transfusion than the non-hyperbilirubinemia group (all *p* < 0.05).

### 3.3. Postoperative Outcomes

Compared with patients without postoperative hyperbilirubinemia, those with hyperbilirubinemia had significantly longer duration of mechanical ventilation and longer ICU stay. They also had higher rates of pneumonia, reintubation, tracheostomy, renal replacement therapy, and in-hospital mortality (all *p* < 0.05). Postoperative hospital length of stay did not differ significantly between the two groups (*p* = 0.095). The postoperative outcomes are summarized in Table 2.

### 3.4. Predictive Value of Peak Total Bilirubin for In-Hospital Mortality

**Figure 2:**
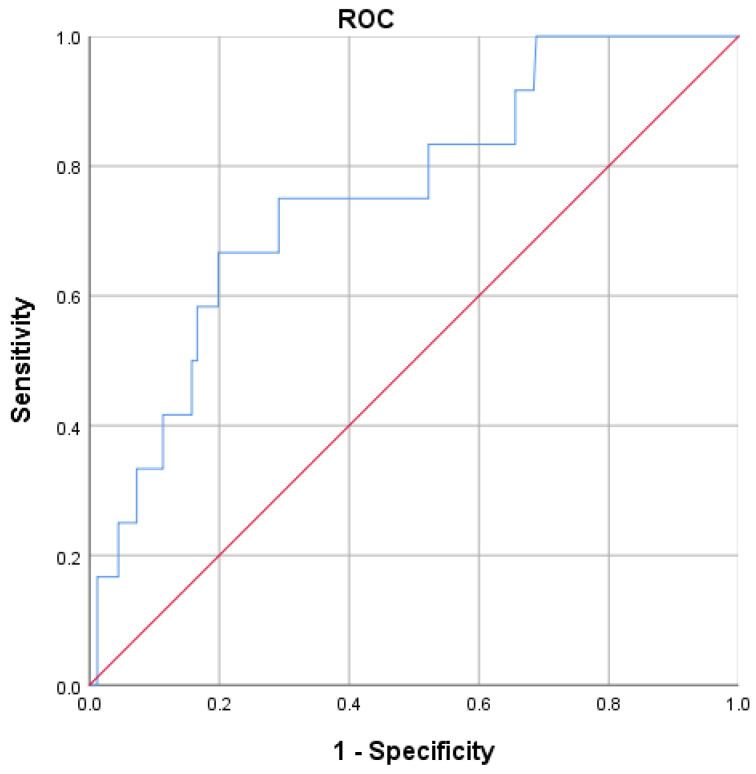
Receiver operator curve (ROC) analysis.

ROC analysis (Figure 2) showed that peak postoperative total bilirubin was associated with increased in-hospital mortality. The optimal cut-off value identified by the Youden index was 3.95 mg/dL, with a sensitivity of 66.7%, specificity of 80.2%, and an area under the curve of 0.756 (*p* = 0.003). This result indicates moderate discriminatory ability, and the cut-off value should be considered exploratory rather than a definitive clinical decision threshold.

### 3.5. Risk Factors for Postoperative Hyperbilirubinemia

Univariable logistic regression analysis showed that higher EuroSCORE II, higher preoperative total bilirubin and direct bilirubin levels, lower hemoglobin and platelet count, pulmonary hypertension, anemia, longer operative time, longer CPB duration, longer aortic cross-clamp time, lower nasopharyngeal temperature, greater blood loss, more red blood cell transfusion, more plasma transfusion, and surgery involving all three valves were associated with postoperative hyperbilirubinemia (all *p* < 0.05) (Table 3).

Because of collinearity among operative time, CPB duration, and cross-clamp time, only CPB duration was entered into the multivariable model. Therefore, we included 13 out of the 15 significant univariate predictors into the multivariate regression model. Multivariable logistic regression analysis demonstrated that higher preoperative direct bilirubin, prolonged CPB duration, and more plasma transfusion were independent risk factors for postoperative hyperbilirubinemia (Table 4). Among patients with preoperative hyperbilirubinemia, 82.93% (34/41) developed postoperative hyperbilirubinemia.

## 4. Discussion

In this retrospective cohort of patients undergoing redo valve surgery, postoperative hyperbilirubinemia occurred in 39.0% of cases and was associated with significantly worse early postoperative outcomes, including prolonged mechanical ventilation, longer ICU stay, higher rates of pulmonary complications and renal replacement therapy, and increased in-hospital mortality. We further identified elevated preoperative direct bilirubin, prolonged CPB duration, and greater plasma transfusion volume as independent risk factors for postoperative hyperbilirubinemia. Importantly, the primary aim of the present study was to identify perioperative factors associated with the development of postoperative hyperbilirubinemia after redo valve surgery. Therefore, the observed relationship between postoperative hyperbilirubinemia and adverse early outcomes should be interpreted as prognostic rather than causal.

Because the mechanisms, disease severity, surgical procedures, and diagnostic thresholds differ across studies, the reported incidence of postoperative hyperbilirubinemia after cardiac surgery varies substantially [9,10,11]. Previous studies have shown that postoperative hyperbilirubinemia is more common after valve surgery than after coronary artery bypass grafting or surgery for congenital heart disease [8,12,13]. In the present study, the incidence was 39.0%, which appears higher than that reported in primary valve surgery (18.9%) [9]. This finding is plausible because redo valve surgery usually involves more extensive adhesiolysis, longer operative exposure, more complex reconstruction, and a higher burden of perioperative hemodynamic stress.

Most patients in our cohort showed a predominantly direct bilirubin elevation, whereas isolated indirect hyperbilirubinemia was uncommon. This pattern suggests that postoperative cholestasis and hepatocellular excretory dysfunction may be more important than overt hemolysis in this population. Previous studies have similarly reported that conjugated hyperbilirubinemia is the dominant phenotype after cardiac surgery and is associated with worse clinical outcomes [9,10,14]. In our cohort, most patients with hyperbilirubinemia reached peak bilirubin levels within the first two postoperative days, and bilirubin levels tended to improve spontaneously once hemodynamics stabilized and oxygen delivery was adequate. This temporal pattern is broadly consistent with previous reports [8]. Yong et al. [12] reported higher mortality in patients whose bilirubin peaked on postoperative day 7 than in those peaking within the first 2 days, but we did not observe a clear difference, possibly because the number of deaths in our study was relatively small.

Our results also confirm the close relationship between postoperative hyperbilirubinemia and adverse postoperative outcomes. Previous studies in cardiac surgery have shown that bilirubin elevation is associated with worse outcomes, especially in patients undergoing complex procedures or prolonged CPB [8,9,11]. We extended these findings to the population undergoing redo valve surgery, a particularly high-risk subgroup. Patients with postoperative hyperbilirubinemia in our study had longer mechanical ventilation and ICU stay, and were more likely to develop pneumonia, require reintubation or tracheostomy, and undergo CRRT. In-hospital mortality was also markedly higher in the hyperbilirubinemia group (8.91% vs. 1.90%).

The association between postoperative hyperbilirubinemia and poor early outcomes is likely multifactorial. Although experimental studies suggest that markedly elevated bilirubin may exert cytotoxic effects under certain conditions [6,15,16,17], our retrospective study does not support a causal interpretation. In the clinical setting of redo valve surgery, postoperative bilirubin elevation is more likely to reflect a combination of perioperative stressors, including impaired hepatic perfusion, venous congestion, cholestatic dysfunction, inflammatory activation, hemolysis, and low cardiac output states. Therefore, postoperative hyperbilirubinemia in our cohort should be interpreted primarily as a clinically meaningful marker of perioperative multisystem stress and hepatic vulnerability rather than a direct mediator of postoperative complications. Another finding of our study is the prognostic value of peak total bilirubin. ROC analysis showed that peak postoperative total bilirubin had moderate discriminatory ability for in-hospital mortality, with an optimal cut-off value of 3.95 mg/dL. Previous studies have also suggested that postoperative bilirubin level may be useful for risk stratification after cardiac surgery, although reported thresholds have differed across cohorts and procedures [11,18]. Such variability is predictable because postoperative bilirubin kinetics may be influenced by surgical complexity, baseline liver status and timing of measurement. Nevertheless, this threshold should be viewed as exploratory and hypothesis-generating rather than as a stand-alone clinical decision point. Clinically, bilirubin should not be used in isolation, but rather interpreted together with hemodynamic variables, renal function, infection markers, respiratory status, and evidence of low cardiac output.

Among the independent predictors identified in our analysis, preoperative direct bilirubin deserves particular attention. Most patients with preoperative bilirubin elevation remained hyperbilirubinemia after surgery, suggesting that impaired preoperative hepatobiliary function may play a central role in the postoperative bilirubin response. Elevated preoperative direct bilirubin may reflect limited hepatic reserve, subclinical cholestasis, or chronic hepatic congestion [16]. In patients with longstanding valvular heart disease, especially those with pulmonary hypertension, tricuspid valve disease, or right-sided dysfunction, chronic venous congestion may lead to congestive hepatopathy and reduced hepatic reserve [19,20,21]. Under the additional stress of redo surgery, these patients may be especially vulnerable to postoperative bilirubin elevation.

We also found that prolonged CPB duration was independently associated with postoperative hyperbilirubinemia. CPB duration likely reflects both operative complexity and exposure to non-physiologic perfusion. Previous studies have shown that CPB can profoundly alter hepatic pathophysiology [12]. Hepatic blood flow may decrease significantly after the initiation of CPB [22], and catecholamine release can further reduce hepatic perfusion and arterial flow [23]. In addition, blood contact with the CPB circuit can trigger inflammatory activation and increase circulating proinflammatory cytokines, contributing to hepatocellular injury. In redo valve surgery, prolonged CPB is often difficult to avoid because of re-entry challenges, extensive adhesiolysis, and complex valve reconstruction. Our findings therefore underscore the importance of meticulous operative planning and technical efficiency to minimize CPB duration whenever feasible.

In univariable analysis, greater intraoperative blood loss and larger red blood cell and plasma transfusion volumes were all associated with postoperative hyperbilirubinemia. Excessive blood loss may indicate perioperative circulatory instability and reduced systemic oxygen delivery, which in turn can impair hepatic and biliary perfusion [24]. Blood transfusion may also increase bilirubin load through hemolysis and reflect a more severe perioperative condition [25]. Interestingly, in multivariable analysis, plasma transfusion volume remained independently associated with hyperbilirubinemia, whereas red blood cell transfusion did not. Rather than indicating a direct causal effect, this association likely reflects perioperative severity, including coagulopathy, bleeding burden, and surgical complexity. Accordingly, plasma transfusion should be interpreted primarily as a marker of a more complicated perioperative course. These findings support the importance of meticulous hemostasis, blood conservation strategies, and goal-directed transfusion management during redo valve surgery.

Our study has several potential clinical implications. First, preoperative direct bilirubin may help identify patients with limited hepatobiliary reserve or chronic congestion who may merit closer perioperative assessment and optimization of anemia, renal function, volume status, and overall physiological reserve. Second, intraoperative strategies aimed at minimizing CPB duration when feasible, maintaining adequate perfusion, and applying careful hemostatic and goal-directed transfusion management may be reasonable. Third, postoperative bilirubin elevation—especially when persistent or accompanied by worsening renal, respiratory, or hemodynamic status—should prompt early reassessment for low cardiac output, venous congestion, bleeding/coagulopathy, or infection. However, these implications should be considered hypothesis-generating, and bilirubin should be used as part of a broader clinical assessment rather than as an isolated trigger for intervention [26,27].

## 5. Limitations

This study has several limitations. First, it was a retrospective single-center study and is therefore subject to selection bias. Second, although multivariable logistic regression was performed, residual confounding cannot be excluded. In particular, detailed intraoperative hemodynamic fluctuations, vasoactive support, right ventricular function parameters, the severity of postoperative low cardiac output syndrome, and infection-related severity were not fully captured in the model. Therefore, the final model may be under-adjusted, and the observed associations should not be interpreted as causal. Third, the sample size was relatively limited, especially for mortality-related analyses. Finally, only in-hospital outcomes were assessed, and the long-term prognostic significance of postoperative hyperbilirubinemia remains unclear.

## 6. Conclusions

Postoperative hyperbilirubinemia is common after redo valve surgery and is significantly associated with worse early postoperative outcomes and higher in-hospital mortality. Elevated preoperative direct bilirubin, prolonged CPB duration, and greater plasma transfusion were independently associated with the development of postoperative hyperbilirubinemia. These findings support the role of bilirubin as a practical marker for perioperative risk stratification in this high-risk surgical population, but it should be interpreted primarily as a marker of perioperative stress and hepatic vulnerability rather than a direct causal determinant of poor outcomes.

## Figures and Tables

**Table 1:** Baseline characteristics of patients undergoing redo valve surgery.

Characteristics	All Cases (*n* = 259)	Hyperbilirubinemia Group (*n* = 101)	Non-Hyperbilirubinemia Group (*n* = 158)	*p*
**Baseline demographics**				
Age, y	57.00 (48.00, 66.00)	57.00 (49.00, 67.00)	57.00 (47.00, 66.00)	0.696
Female sex, *n* (%)	160 (61.78)	60 (59.41)	100 (63.29)	0.530
BMI, kg/m^2^	21.60 (19.65, 23.88)	21.51 (19.53, 23.87)	21.56 (19.72, 23.69)	0.764
Interval, y	15.00 (10.00, 23.00)	15.00 (10.00, 24.00)	16.00 (9.00, 22.25)	0.530
EuroSCORE II, (%)	7.30 (4.58, 11.39)	9.06 (6.45, 12.99)	6.56 (4.02, 10.19)	<0.001
**Comorbidities**				
Diabetes (type 1 or 2), *n* (%)	22 (8.49)	7 (6.93)	15 (9.49)	0.471
Smoking, *n* (%)	24 (9.27)	7 (6.93)	17 (10.76)	0.300
Hypertension, *n* (%)	33 (12.74)	11 (10.89)	22 (13.92)	0.475
Pulmonary hypertension, *n* (%)	210 (81.08)	91 (90.10)	119 (75.32)	0.003
Preoperative atrial fibrillation, *n* (%)	171 (66.02)	70 (69.31)	101 (63.92)	0.372
Anemia, *n* (%)	139 (53.67)	65 (64.36)	74 (46.84)	0.006
Infectious endocarditis (active), *n* (%)	17 (6.56)	3 (2.97)	14 (8.86)	0.062
**Cardiac function**				
LVEF, %	63.00 (56.00, 70.00)	63.00 (56.00, 70.00)	63.00 (56.45, 70.00)	0.891
NYHA-3,4, *n* (%)	202 (77.99)	82 (81.19)	120 (75.95)	0.321
NYHA-2, *n* (%)	57 (22.01)	19 (18.81)	38 (24.05)	0.321
**Preoperative labs**				
Preoperative eGFR, ml/min/1.73 m^2^	85.12 (64.11, 99.88)	82.95 (60.86, 96.57)	86.30 (66.89, 101.43)	0.083
Baseline serum creatinine, μmol/L	74.00 (61.00, 95.00)	78.00 (63.50, 101.00)	72.50 (59.75, 87.25)	0.064
Preoperative albumin, g/L	40.00 (37.30, 42.20)	40.00 (37.20, 41.95)	40.30 (37.35, 42.20)	0.689
Preoperative total bilirubin, μmol/L	19.00 (13.40, 29.80)	28.80 (20.80, 44.95)	15.90 (10.65, 22.20)	<0.001
Preoperative direct bilirubin, μmol/L	7.90 (5.30, 12.80)	12.40 (9.00, 20.95)	5.85 (4.55, 9.03)	<0.001
Preoperative alanine aminotransferase, U/L	16.00 (12.00, 23.00)	16.00 (13.00, 23.00)	15.50 (12.00, 23.00)	0.762
Preoperative aspartate aminotransferase, U/L	26.00 (21.00, 36.00)	30.00 (22.00, 42.50)	25.00 (20.75, 33.00)	0.009
Hemoglobin, g/L	116.00 (99.00, 130.00)	109.43 ± 27.58	116.87 ± 22.30	0.014
White blood cell, 10^9^/L	4.87 (3.76, 6.41)	4.82 (3.66, 6.62)	4.95 (3.96, 6.20)	0.482
Platelet, 10^9^/L	162.74 ± 60.00	151.49 ± 54.20	169.94 ± 62.54	0.015
**Operative variables**				
Operation time, h	6.42 (5.20, 7.75)	6.87 (5.93, 8.80)	5.87 (4.91, 7.09)	<0.001
CPB duration, h	3.18 (2.33, 4.22)	3.77 (2.81, 4.88)	2.68 (2.07, 3.83)	<0.001
Cross-clamp time, h	2.18 (1.50, 2.88)	2.47 (1.84, 3.13)	1.93 (1.38, 2.79)	<0.001
Minimum nasopharyngeal temperature, °C	30.80 (30.00, 31.40)	30.50 (30.00, 31.00)	30.90 (30.00, 31.50)	0.053
CPB-MAP, mmHg	53.72 ± 5.75	53.75 ± 6.35	53.71 ± 5.35	0.955
Type of surgery				
Single aortic valve, *n* (%)	10 (3.86)	3 (2.97)	7 (4.43)	0.552
Single mitral valve, *n* (%)	33 (12.74)	5 (4.95)	28 (17.72)	0.003
Single tricuspid valve, *n* (%)	36 (13.90)	9 (8.91)	27 (17.09)	0.064
Aortic valve + mitral valve, *n* (%)	16 (6.18)	6 (5.94)	10 (6.33)	0.899
Aortic valve + tricuspid valve, *n* (%)	24 (9.27)	9 (8.91)	15 (9.49)	0.875
Mitral valve + tricuspid valve, *n* (%)	71 (27.41)	31 (30.69)	40 (25.32)	0.344
Aortic valve + mitral valve + tricuspid valve, *n* (%)	69 (26.64)	38 (37.62)	31 (19.62)	0.001
Valve + CABG, *n* (%)	7 (2.70)	3 (2.97)	4 (2.53)	0.832
Intraoperative blood loss, mL	700.00 (500.00, 1100.00)	800.00 (575.00, 1500.00)	600.00 (445.00, 900.00)	<0.001
Red blood cells transfusion, U	4.00 (2.00, 8.00)	4.00 (2.00, 9.00)	4.00 (0.00, 6.00)	0.001
Plasma transfusion, mL	400.00 (200.00, 600.00)	600.00 (400.00, 800.00)	400 (0.00, 400.00)	<0.001
Platelet transfusion, U	1.00 (0.00, 1.00)	1.00 (0.00, 1.00)	1.00 (0.00, 1.00)	0.313

BMI, body mass index; LVEF, left ventricular ejection fraction; NYHA, New York Heart Association (functional classification); EuroSCORE, European System for Cardiac Operative Risk Evaluation; eGFR, estimated glomerular filtration rate; CPB, cardiopulmonary bypass; MAP, mean arterial pressure; CABG, coronary artery bypass grafting.

**Table 2:** The major outcomes of the included patients.

Characteristics	Hyperbilirubinemia Group (*n* = 101)	Non-Hyperbilirubinemia Group (*n* = 158)	*p*
Mechanical ventilation, h	22.00 (17.50, 61.50)	19.00 (17.00, 24.50)	0.002
ICU stay, d	4.00 (3.00, 6.00)	3.00 (2.00, 4.00)	<0.001
Postoperative hospital stay, d	12.00 (9.00, 18.00)	11.00 (8.00, 17.00)	0.095
Pneumonia, *n* (%)	8.00 (7.92)	3.00 (1.90)	0.019
Re-intubation, *n* (%)	12.00 (11.88)	2.00 (1.27)	<0.001
Use of tracheotomy, *n* (%)	7.00 (6.93)	1.00 (0.63)	0.004
CRRT, *n* (%)	14.00 (13.86)	7.00 (4.43)	0.007
Use of ECMO, *n* (%)	0.00 (0.00)	1.00 (0.63)	0.423
Hospital mortality, *n* (%)	9.00 (8.91)	3.00 (1.90)	0.009

ICU, intensive care unit; CRRT, continuous renal replacement therapy; ECMO, extracorporeal membrane oxygenation.

**Table 3:** Univariate analysis of factors associated with postoperative hyperbilirubinemia.

Variable	Odds Ratio	95% CI	*p*
**Preoperative variables**			
EuroSCORE II	1.042	(1.005–1.081)	0.026
Preoperative total bilirubin	1.108	(1.076–1.141)	<0.001
Preoperative direct bilirubin	1.259	(1.179–1.346)	<0.001
Hemoglobin	0.987	(0.977–0.998)	0.019
Pulmonary hypertension	2.829	(1.337–5.990)	0.007
Anemia	2.050	(1.227–3.424)	0.006
Platelet	0.995	(0.990–0.999)	0.017
**Intraoperative variables**			
Operation time	1.424	(1.231–1.646)	<0.001
CPB duration	1.601	(1.315–1.948)	<0.001
Cross-clamp time	1.615	(1.255–2.080)	<0.001
Minimum nasopharyngeal temperature	0.783	(0.626–0.979)	0.032
Intraoperative blood loss *	1.070	(1.031–1.110)	<0.001
Red blood cells transfusion	1.122	(1.058–1.190)	<0.001
Plasma transfusion *	1.194	(1.105–1.290)	<0.001
Aortic valve + mitral valve + tricuspid valve	2.471	(1.408–4.336)	0.002

CI, confidence interval; EuroSCORE, European System for Cardiac Operative Risk Evaluation; CPB, cardiopulmonary bypass. * Odds ratios for blood loss and plasma transfusion were expressed per 100 mL increment for easier interpretation.

**Table 4:** Multivariable analysis of factors associated with postoperative hyperbilirubinemia.

Variable	Odds Ratio	95% CI	*p*
Preoperative direct bilirubin	1.243	1.161–1.329	<0.001
CPB duration	1.482	1.173–1.870	0.001
Plasma transfusion *	1.144	1.041–1.257	0.005

CI, confidence interval; CPB, cardiopulmonary bypass. * Odds ratios for plasma transfusion were expressed per 100 mL increment for easier interpretation.

## Data Availability

The original contributions presented in the study are included in the article, and further inquiries can be directed to the corresponding author.
